# Air pollution and migraine: causal association or epiphenomenon?

**DOI:** 10.1097/MD.0000000000047274

**Published:** 2026-02-20

**Authors:** Qian Zhu, Jin Yang, Lei Shi, Jieying Zhang, Xiaohu Zhang, Lina Ma, Gang Huang, Xuancheng Zhou, Xiaoli Song

**Affiliations:** a Department of Acupuncture, First Teaching Hospital of Tianjin University of Traditional Chinese Medicine, Tianjin, China; b Department of Acupuncture, National Clinical Research Center for Chinese Medicine, Tianjin, China; c Clinical Medical College, Southwest Medical University, Luzhou, China.

**Keywords:** air pollution, migraine, nitrogen oxides, particulate matter, 2-sample Mendelian randomization

## Abstract

Migraine is a common chronic neurological disorder that poses a significant burden to global health. Investigating the risk factors for migraine is crucial for its prevention and management. Air pollution, one of the greatest environmental risks to public health today, has not yet been conclusively linked to migraine, and the quality of existing studies varies widely. To avoid potential confounders in observational studies, this research utilizes the 2-sample Mendelian randomization (MR) approach for the first time to reassess the causal relationship between air pollution indicators such as PM2.5, PM10, PM2.5 to 10, nitrogen dioxide (NO2), and nitrogen oxides (NOx), and migraine. This study employs a 2-sample MR design, utilizing genome-wide association summary data on air pollution and migraine from the IEU and FinnGen databases. The primary method used is inverse-variance weighting, combined with 4 other MR analysis techniques, to thoroughly investigate the data. Concurrently, a series of sensitivity analyses are conducted using Cochran *Q* test, MR-Egger intercept regression, and MR-PRESSO. The combined MR and sensitivity analysis of various air pollutants and migraine showed a high degree of robustness across all data except for the PM10 results. After applying the Bonferroni correction, all MR analysis results did not reach the significance threshold for p-values, indicating no statistically significant causal relationship between air pollution and migraine. This study did not find a significant causal relationship between air pollution and migraine. Combining previous studies, we speculated on the potential reasons behind our analysis results. Considering the limitations of this study, we recommend further research to validate our analysis results and to explore this topic more deeply.

## 1. Introduction

Migraine is a chronic neurological disorder that frequently recurs, often accompanied by symptoms of nausea and vomiting, and in certain cases, by photophobia, phonophobia, anxiety, and depression. A 2021 study on global disease burden found that migraine ranks third in age-standardized disability-adjusted life years among all neurological disorders.^[1–3]^ A cross-sectional study from Europe indicates that migraine has become the leading cause of disability in people under 50.^[4]^ Despite increasing research on migraines in recent years, the specific triggers and mechanisms of migraines remain unclear. Environmental factors have been considered one of the triggers for the onset and recurrence of migraines in previous studies.^[5]^

Air pollution has been defined by the World Health Organization as one of the greatest environmental risks to public health, posing a significant threat globally.^[6]^ A 2019 global disease burden study revealed that air pollution is the fourth leading risk factor for premature death worldwide, following unhealthy diets, smoking, and high blood pressure.^[7]^ Over the past few decades, with increased awareness and technological advances in developed countries, air pollution has been somewhat controlled and improved, yet it remains a significant threat to human health.^[8]^ Air pollution is a complex mixture of chemical and environmental elements, consisting of particulates, gases, dust, fumes, and biological materials. The most common particulate pollutants are PM2.5 and PM10, and the most common gaseous pollutants include carbon monoxide (CO), NOX, ozone, and sulfur dioxide.^[6]^ These chemical particles and gases vary in their sources across space and time, as well as in their physical and chemical properties and toxicity.^[9]^

In past epidemiological studies on air pollution and migraines, air pollution has been identified as a significant risk factor for migraines. For instance, a systematic review studying the association between environmental air pollution and migraines suggested that exposure to NO2, CO, PM10, and PM2.5 is associated with migraines^[10]^; A statistical analysis of emergency department records for urban residents in Canada shows that environmental air pollution is associated with an increase in visits for neurological diseases, with a majority being for migraines, accounting for 39%.^[11]^ On the other hand, some studies find no significant association between air pollution and migraines. For example, a systematic review of 12 studies found that most research on the relationship between air pollution and migraines carries a low to moderate risk of bias, with higher-quality articles typically reporting a weaker association.^[12]^ In summary, there remains controversy over the impact of air pollution on migraines. Traditional research methods struggle to control for confounding factors that bias the analysis, necessitating further research into the association between air pollution and migraines.

Mendelian randomization (MR) is an effective tool for identifying causal relationships between exposures and outcomes.^[13–16]^ According to Mendel laws of genetics, genetic variants are distributed evenly, randomly, and independently during meiosis. The instrumental variables (IVs) used in MR are genetic variants, effectively avoiding the effects of confounders and reverse causation on the true causal relationship.^[17]^ In past genome-wide association studies, thousands of genetic variants have been identified as associated with various complex diseases, laying the groundwork for the widespread application of MR.^[18]^ This article builds on the aforementioned knowledge, using a 2-sample MR analysis of recent large-scale GWAS summary data to explore the potential association between air pollution and migraines.

## 2. Materials and methods

### 2.1. Study design

**Figure 1.**
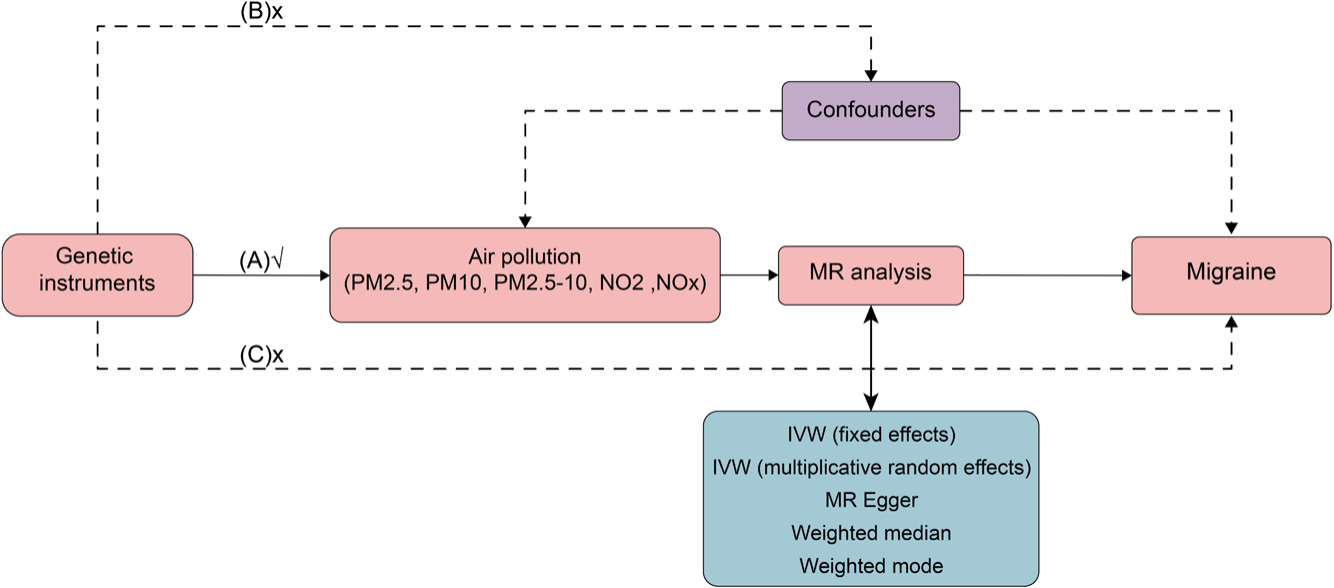
Overview of the flowchart assumptions and design scheme. SNPs related to air pollutants (PM2.5, PM10, PM2.5–10, NO_2_, NO_X_) are used as genetic tools to study the causal impact of air pollution on migraines. (A, B) C represent the 3 core hypotheses: (A) Arrows indicate that the genetic tools (SNPs) are associated with exposure and affect the outcome only through exposure. (B and C) Dashed lines indicate that the genetic tools (SNPs) are unrelated to confounding factors between the genetic tools and the outcome. IVW = inverse variance weighting, MR = Mendelian randomization, NO_2_ = nitrogen dioxide, NO_X_ = nitrogen oxides, PM = particulate matter, SNPs = single nucleotide polymorphisms.

This MR study was rigorously conducted in accordance with the STROBE-MR guidelines.^[19]^ The MR analysis process is shown in Figure 1. We use single nucleotide polymorphisms (SNPs) in genetic variations as IVs to reassess the impact of air pollution on migraines. It is noteworthy that all MR analyses adhere to 3 core assumptions: The relevance assumption: IVs are strongly associated with the exposure; the independence assumption: IVs are independent of any confounders, thereby excluding confounder effects; the exclusion restriction assumption: IVs affect the outcome solely through the exposure, without any direct correlation to the outcome.^[20]^ Based on these core assumptions, we selected IVs that best represent air pollution (PM2.5, PM2.5–10, PM10, nitrogen dioxide, and NOX) to evaluate their effects on migraines. The data used in the analyses were sourced from recent large-scale GWAS summary data on European populations, publicly available on the MRC-IEU and FinnGen websites.

### 2.2. Data source

The detailed information about the data sources is shown in Table 1. We obtained GWAS summary datasets on air pollution (PM2.5, PM2.5–10, PM10, NO2, and NOX) from the IEU Open GWAS database (https://opengwas.io). These data are outputs from a GWAS pipeline using Phesant-derived variables from the UKBiobank, conducted by Ben Elsworth and colleagues.^[21]^ The original study by the European Study of Cohorts for Air Pollution Effects assessed the annual average concentrations of PM2.5, PM10, PM2.5 to 10, NO2, and NOx in 20 European regions from January 26, 2010, to January 18, 2011, using a land use regression model (LUR).^[22,23]^

**Table 1:** Summary of the GWAS included in this 2-sample MR study.

Trait		Sex	Sample size	Consortium	GWAS ID	Population	Year
Particulate matter (PM)	PM2.5	Males and Females	423,796	MRC-IEU	ukb-b-10817	European	2010
	PM10	Males and Females	455,314	MRC-IEU	ukb-b-589	European	2010
	PM2.5–10	Males and Females	423,796	MRC-IEU	ukb-b-12963	European	2010
Nitrogen dioxide		Males and Females	456,380	MRC-IEU	ukb-b-2618	European	2010
Nitrogen oxides		Males and Females	456,380	MRC-IEU	ukb-b-12,417	European	2010
Migraine		Males and Females	520,210	FinnGen	finngen_R9_MIGRAINE_TRIPTAN	European	2019

GWAS = genome-wide association studies, MR = Mendelian randomization, PM = particulate matter, SNPs = single nucleotide polymorphisms.

The GWAS summary data for migraines in this study come from the FinnGen study, a large-scale genomic study within Finland involving over 500,000 samples, which analyzes the association between genetic variants and health data to explore disease mechanisms.^[24]^ Migraine is a common and severe vascular headache, often associated with increased sympathetic nerve activity, leading to nausea, vomiting, and photosensitivity. The migraine data GWAS ID includes 57,223 cases and 520,210 controls. In the FinnGen database, migraine is defined as requiring the purchase of a specific triptan medication and having an ICD code. It is worth noting that all data in this study are from publicly available GWAS studies on European populations, thus posing no ethical issues. Furthermore, the data on air pollution and migraines come from 2 different GWAS studies, with no known overlap between the samples.

### 2.3. Selection of instrumental variables

For the selection of IVs, aiming to enhance statistical efficiency while maintaining the 3 fundamental assumptions of MR, we conducted rigorous filtering steps. Initially, to ensure a strong correlation between IVs and exposures, not all data could select enough SNPs for analysis at a significance threshold of 5E-8. To maximize the number of IVs while maintaining statistical power, we set specific thresholds for SNPs associated with each air pollutant. 5E-8 was used as the threshold for filtering PM10-associated SNPs, 5E-7 for PM2.5, NO2, and NOX -associated SNPs, and 5E-6 for PM2.5 to PM10. Next, to ensure a robust correlation between IVs and the corresponding air pollutants, we quantified the strength of each SNP using the F-statistic, calculated as *F* = (β/SE[β])^2^.^[25]^ Only SNPs with an *F*-statistic >10 were retained for further analysis to eliminate biases introduced by weak IVs. Additionally, using the “clump_data” function in the TwoSampleMR package, we removed linkage disequilibrium SNPs between IVs online, setting parameters to *R*^2^ <0.001, kb >10,000kb.^[26]^ Subsequently, using the PheLiGe (https://phelige.com) tool, we individually screened the remaining SNPs to exclude those related to outcomes and confounding factors (*P* <5 × 10^−08^, *R*^2^ >0.8), to eliminate the pleiotropy of SNPs affecting the MR results.^[27]^ Then, palindromic SNPs were excluded to ensure that SNPs in the exposure and outcome datasets corresponded to the same loci. Finally, to further address horizontal pleiotropy in MR analysis, outlier SNPs were detected and excluded using MR-PRESSO.

### 2.4. Mendelian randomization analysis

In this study, we used 5 methods to reassess the effects of various air pollutants on migraine. The inverse-variance weighted (IVW) fixed and random effects models were used as primary outcomes to reassess the effects of air pollutants on migraine.^[28,29]^ Additionally, supplementary methods like MR-Egger,^[30]^ weighted median^[31]^ and weighted model^[32]^ served as auxiliary references to enhance the precision and reliability of the MR analysis. During the analysis, for the IVW and MR-Egger methods, Cochran *Q* test was used to check for heterogeneity among IVs.^[33]^ If the test result *P* <.05 indicated significant heterogeneity, the IVW random effects model was selected as the final outcome; otherwise, the IVW fixed effects model was used. It is worth noting that regardless of the MR analysis outcomes, the effect estimates from other methods should be consistent with those from the IVW method; otherwise, the results are considered not to have significant statistical meaning.^[34]^

### 2.5. Sensitivity analysis

To further assess the robustness of the analysis results, we conducted systematic sensitivity analyses. Initially, we used MR-Egger intercept regression to test whether SNPs in the IVs exhibit horizontal pleiotropy and if it impacts the results.^[35]^ If the MR-Egger intercept regression analysis shows an intercept near zero and *P* >.05, it indicates no horizontal pleiotropy in SNPs within IVs. We used the leave-one-out method to test for heterogeneity in SNPs within IVs. By removing each SNP individually and recalculating the overall effect size, we assessed whether the MR analysis results were biased by highly heterogeneous SNPs.^[36]^ Additionally, scatter plots, forest plots, and funnel plots were used to visualize the MR analysis results, checking for the presence of high-influence point SNPs. At the same time, to avoid horizontal pleiotropy in SNPs within IVs, we conducted a global test for MR outliers using the MR-PRESSO package. If the global test *P* <.05 indicates horizontal pleiotropy, outlier SNPs were identified and removed through MR-PRESSO outlier test function.^[37]^

### 2.6. Statistical analysis

The packages used in the statistical analysis of this study were “TwoSampleMR” and “MR-PRESSO.” All statistical analyses were performed using version 4.3.2 of the R software. The *P*-values for the MR analysis results were Bonferroni-adjusted: *P* = .05/5 = 0.01, and all statistical tests were 2-sided.

## 3. Result

### 3.1. Strength of genetic instruments

**Figure 2.**
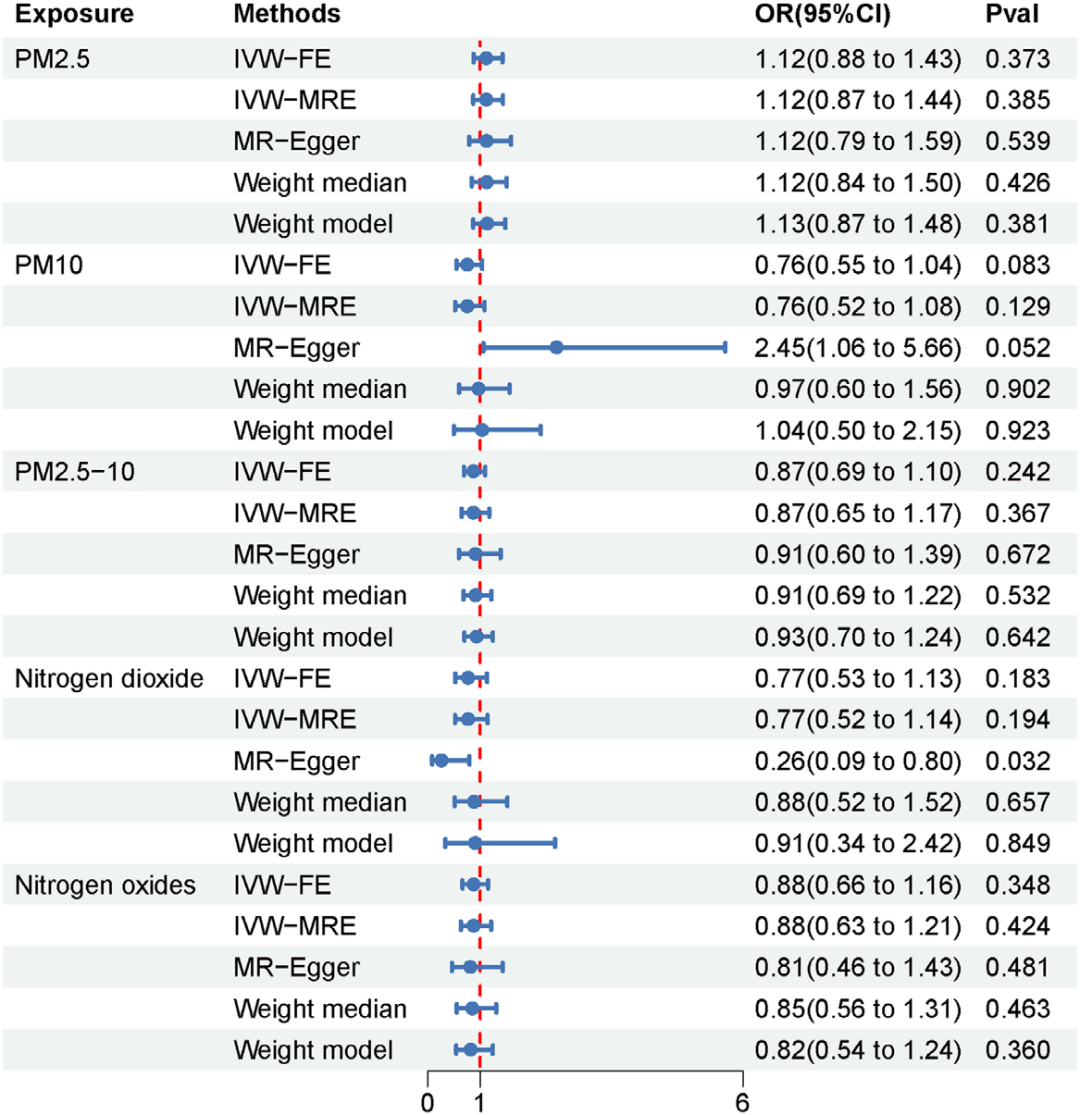
MR analysis of air pollution (particulate matter, nitrogen dioxide, and nitrogen oxides) with migraine outcome in a European population. The red line indicates the reference OR of 1, while blue lines represent the 95% CIs for each exposure. 95% CI = 95% confidence interval, IVW = inverse-variance weighted, OR = odds ratio, MR = Mendelian randomization, PM = particulate matter.

In this study, the number of SNPs corresponding to the MR analysis of various air pollutants and migraine is shown in Figure 2. Details on the selection of IVs are provided in Table S1–5, Supplemental Digital Content, https://links.lww.com/MD/R315. The statistical calculations revealed that the F-statistics for all IVs were >20. Furthermore, there are no known overlaps between the GWAS cohorts for various air pollutants and those for migraine, indicating that the selected IVs effectively avoid biases introduced by potential weak IVs. Simultaneously, outlier SNPs were identified and removed using MR-PRESSO, followed by a global test estimation. Lastly, the MR-PRESSO global test results for all air pollutants corresponding to IVs were >0.05 (Table S6, Supplemental Digital Content, https://links.lww.com/MD/R315), indicating that MR-PRESSO did not detect any horizontal pleiotropy in the IVs selected for MR analysis (Fig. 2).

### 3.2. The impact of air pollution on migraines

**Figure 3.**
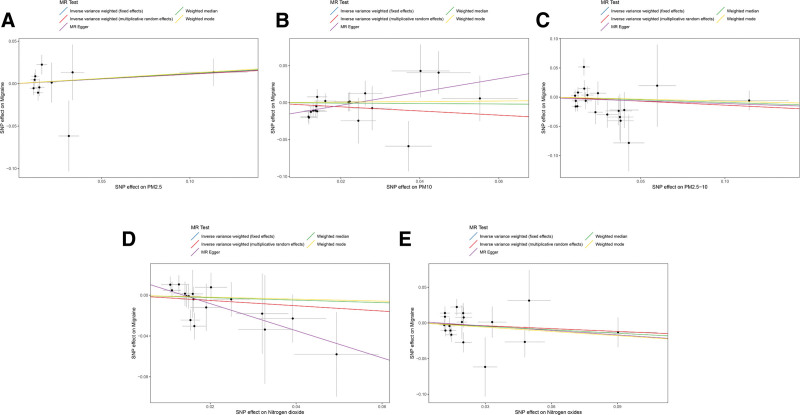
Scatter plot of the estimated effects of air pollution (particulate matter, nitrogen dioxide, and nitrogen oxides) on migraine in the European population through SNPs. Each black dot represents an individual SNP, plotted with error bars corresponding to each SE. The slope of each line corresponds to the combined estimates from methods using the weighted fixed effects model of inverse variance (light blue line), weighted random effects model of inverse variance (red line), MR-Egger (purple line), weighted median (green line), and weighted mode (yellow line). (A) PM2.5; (B) PM10; (C) PM2.5 to 10; (D) nitrogen dioxide; (E) nitrogen oxides. SE = standard error, SNPs = single nucleotide polymorphisms.

**Figure 4.**
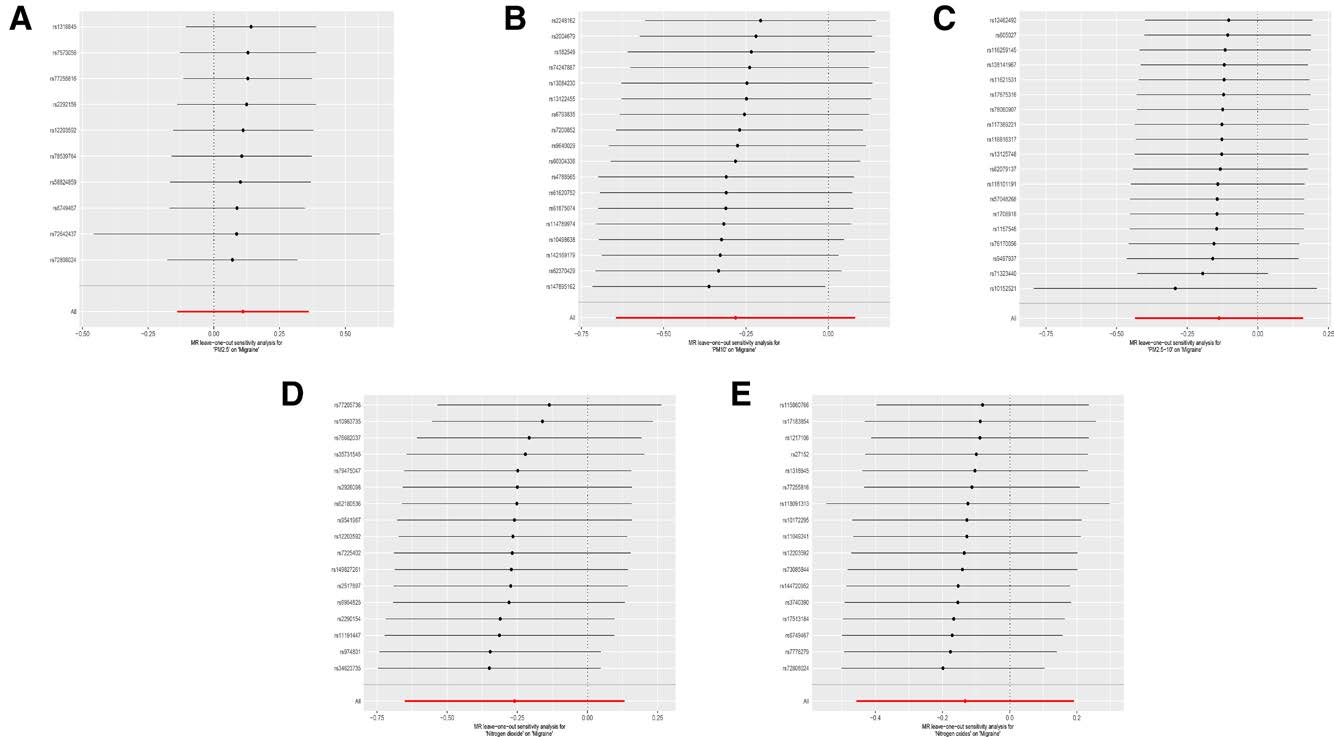
Forest plot of the causal SNP effects of air pollution (particulate matter, nitrogen dioxide, and nitrogen oxides) on migraine in the European population. Error bars represent the 95% CI. (A) PM2.5; (B) PM10; (C) PM2.5 to 10; (D) nitrogen dioxide; (E) nitrogen oxides. CI = confidence interval, SNPs = single nucleotide polymorphisms.

To explore the effects of air pollution on migraine, we conducted MR analyses for common air pollutants including PM2.5, PM10, PM2.5 to 10, NO_2_, and NO_X_ against migraine. The results of all MR analyses are summarized in Figure 2. We visualized the effects of air pollutants on migraine assessed by 5 methods using scatter plots (Fig. 3). Sensitivity analyses of MR results for PM2.5, NO_2_, and NO_X_ with migraine showed that Cochran *Q* tests were all *P* >.05, and funnel plot visualizations as in Figure S1, Supplemental Digital Content, https://links.lww.com/MD/R315 revealed no heterogeneity, hence the IVW fixed effect model was chosen as the primary outcome; Both MR-Egger intercept regression tests and MR-PRESSO global tests were *P* >.05, indicating no horizontal pleiotropy detected in MR analysis results; The leave-one-out sensitivity test as shown in Figure 4 revealed no significant impact of any single SNP on the overall effect estimate. However, the main results from the IVW fixed model and other methods used as auxiliary references all had *P* >.01, indicating no significant statistical meaning. It is noteworthy that in the MR analysis between PM10 and migraine, the MR-Egger intercept regression result was *P* <.05, indicating that MR-Egger detected potential horizontal pleiotropy in IVs. Using the PheLiGe tool to individually screen SNPs, no SNPs related to potential confounders were found. Moreover, the MR-PRESSO global test results were *P* >.05, with no SNPs exhibiting horizontal pleiotropy found. To ensure the robustness of the conclusions, we consider the MR analysis between PM10 and migraine to be not informative. Additionally, in the MR analysis of PM2.5 to 10 with migraine, Cochran Q test results were *P* <.05, indicating heterogeneity, thus the IVW random effect model was chosen as the main outcome. In this MR analysis, both MR-PRESSO and MR-Egger intercept regression had *P* >.05, indicating no significant horizontal pleiotropy; The leave-one-out sensitivity test visualization as shown in Figure 4 found no significant effects of any single SNP on the overall effect estimate. However, all MR analysis results were *P* >.01, indicating no significant impact of PM2.5 to 10 on migraine. In summary, combining all MR analysis results of air pollutants with migraine in this study, there is no evidence to suggest that air pollution significantly impacts migraine.

## 4. Discussion

This study investigated the effects of air pollution on migraine using 2-sample MR analysis. MR analysis was performed using GWAS summary data for air pollutants PM2.5, PM10, PM2.5 to 10, NO2, and NOX. The analysis results showed that the effects of PM2.5, NO2, and NOX on migraine had *P* >.01, indicating no significant effects, and sensitivity analysis found no anomalies, suggesting reliable results. In the analysis of PM2.5 to 10, Cochran Q test for heterogeneity was *P* <.05, indicating the presence of heterogeneity, thus the IVW random effects model was chosen as the primary result. Notably, in the MR analysis of PM10, the MR-Egger intercept regression was *P* <.05, indicating detected horizontal pleiotropy, but no outlier SNPs were found using the PheLiGe tool or MR-PRESSO outlier tests. Given the aberrant performance of the MR-Egger intercept regression, the MR analysis results for PM10 are considered not to be informative. In summary, this study found no evidence to support that air pollutants (PM2.5, PM10, PM2.5–10, NO2, NOX) have a significant effect on migraine.

Reviewing past studies on the association between air pollution and the impact on migraine. For instance, a case-crossover analysis in Seoul showed that air pollution might trigger migraines, especially in hot weather.^[38]^ Similarly, a case-crossover analysis in Taiwan showed that high levels of air pollutants increase the risk of migraine consultations.^[39]^ However, unlike the above, a systematic review on the impact of short-term air pollution exposure on migraine found that the existing evidence on the association between different air pollutants and migraine is inconsistent, with varying methods and quality, necessitating updated assessment methods for quantitative analysis to reach reliable conclusions.^[40]^ Therefore, in this study, we employed a novel approach, MR analysis, to analyze the impact of air pollution on migraine.

Previous studies on how air pollution affects migraine have proposed hypotheses, such as PM2.5 activating oxidative stress pathways to trigger neurogenic inflammation, mediating a series of biological changes in the nervous system, considered an effective toxic component of PM.^[41,42]^ Additionally, harmful stimuli may trigger responses in different systems, where lesions in the neurological and vascular systems could lead to migraines.^[43]^ For example, the normal dilation of endothelium-dependent vessels might be impaired by air pollutants, leading to migraines.^[44]^ Although many hypotheses have been proposed in past studies to explain the association between air pollution and migraine, whether this association truly exists and the specific mechanisms still require further research and validation. The analysis results of this study did not find a significant association between air pollution and migraine, thus we believe there is a lack of statistical association between air pollution and migraine, refuting the view that air pollution is a cause of migraine. Regarding the effects of air pollution on migraine combined with the interpretation of MR analysis results, we speculate that mere air pollution may not cause migraine; further research should consider the impact of other environmental factors. For instance, studies have shown that higher relative humidity in warmer seasons is associated with a higher incidence of migraine, while gaseous pollutants related to traffic may be linked to a higher incidence of migraine in colder seasons.^[45]^ Factors such as temperature, humidity, and seasonal climate may synergistically act with air pollution as triggers for migraine. Additionally, the duration of exposure to air pollution is also an important factor. A review indicates that short-term exposure to PM2.5 is not necessarily associated with migraine, whereas long-term exposure to PM2.5 is related to the severity and incidence of migraine.^[5]^ More precisely defining the duration of exposure to air pollution would benefit further exploration into the effects of air pollution on human health.

Compared to previous studies, our research has the following relative advantages: This is the first study to explore the association between air pollution and migraine using MR analysis. Traditional research designs and condition controls are often susceptible to confounding factors, leading to variations in quality and conclusions. MR analysis is an efficient and sensitive statistical analysis that uses genetic variants as IVs. Genetic variants are uniformly, randomly, and independently distributed during meiosis, thus effectively avoiding the influence of confounding factors. Additionally, the temporal precedence of genetic variants over the occurrence of disease helps to avoid the impact of reverse causality on effect analysis.^[46]^ The analysis data come from 2 large-scale GWAS studies, with no sample overlap between them, effectively avoiding biases from weak IVs, while the large sample size enhances statistical power. For the MR analysis results, we conducted a series of rigorous sensitivity analyses including MR-Egger intercept regression, Cochran *Q* test, leave-one-out sensitivity analysis, and MR-PRESSO global test, ensuring the robustness of our findings.

Despite these strengths, our study still has certain limitations. In this study, we only used PM2.5, PM10, PM2.5 to 10, NO2, and NOX as representatives of air pollution for MR analysis. In fact, air pollutants are a complex mixture of many harmful substances, and due to data limitations, we did not include other harmful air pollutants for coordinated analysis. Moreover, all the GWAS summary data were based on European populations, so analyses of other populations are needed to better generalize the conclusions. Additionally, the MR approach, while effective for assessing long-term genetic associations, has inherent limitations when evaluating short-term environmental exposures like air pollution. MR relies on genetic variants that reflect lifetime exposure patterns rather than acute or transient changes, potentially underestimating the impact of short-term air pollution spikes on migraine onset. Future studies could complement MR with real-time exposure data or longitudinal designs to better capture these dynamic effects.

## 5. Conclusion

In conclusion, our first study based on a 2-sample MR analysis of a European population does not support a significant association between air pollution and migraine. Regarding the association between air pollution and migraine, our study still has certain limitations, so further research is needed to validate the findings of this study.

## Acknowledgements

At the conclusion of our study, we would like to express our special thanks to all participants and researchers involved in the FinnGen, MRC-IEU, and UKBiobank studies. Without their diligent efforts, this Mendelian randomization study would not have been possible. Additionally, we are grateful to the developers of the TwoSampleMR and MR-PRESSO software packages for providing convenient and efficient tools for our research.

## Author contributions

**Writing – original draft:** Qian Zhu, Jin Yang, Lei Shi, Jieying Zhang, Xiaohu Zhang, Lina Ma, Gang Huang, Xuancheng Zhou, Xiaoli Song.

**Writing – review & editing:** Xiaoli Song.

## Supplementary Material
