# Supplementary material for: Air pollution and migraine: causal association or epiphenomenon?
Source: Medicine (Baltimore). 2026 Feb 20;105(8):e47274. doi: 10.1097/MD.0000000000047274 (PMC12928860; doi:10.1097/MD.0000000000047274)
Supplement: Supplementary file 1 [file medi-105-e47274-s001.pdf]

SNP	other_allele	effect_allele	beta.expos	se.exposure	pval.expos	mr_keep.e	f
rs6749467	G	A	-0.01239	0.002183	1.40E-08	TRUE	32.22849
rs7573056	A	C	-0.01141	0.00217	1.50E-07	TRUE	27.6423
rs1318845	T	C	-0.01397	0.002699	2.30E-07	TRUE	26.77719
rs1372504	G	A	0.012291	0.002219	3.10E-08	TRUE	30.67374
rs58824859	A	C	0.012008	0.002305	1.90E-07	TRUE	27.14069
rs72808024	A	C	-0.01609	0.00302	9.90E-08	TRUE	28.38578
rs12203592	C	T	0.021666	0.002591	6.20E-17	TRUE	69.91813
rs77255816	C	T	0.031394	0.005728	4.20E-08	TRUE	30.04086
rs114708313	A	T	0.024558	0.004478	4.20E-08	TRUE	30.07625
rs77205736	C	T	0.013522	0.002413	2.10E-08	TRUE	31.39913
rs1537371	C	A	0.012371	0.002149	8.50E-09	TRUE	33.14877
rs11042316	G	A	-0.01304	0.002498	1.80E-07	TRUE	27.25365
rs78539764	T	C	-0.03352	0.00659	3.70E-07	TRUE	25.86871
rs11855821	G	A	-0.01262	0.002432	2.10E-07	TRUE	26.959
rs2292156	G	T	0.014681	0.002878	3.40E-07	TRUE	26.02641
rs72642437	C	T	0.113396	0.019135	3.10E-09	TRUE	35.11871

Supplementary Table 1: The instrumental variables of PM2.5

SNP	other_allele	effect_allele	beta.expos	se.exposure	pval.expos	mr_keep.e	f
rs114789974	C	A	-0.05521	0.009635	1.00E-08	TRUE	32.83232
rs182549	C	T	-0.01242	0.002219	2.10E-08	TRUE	31.35735
rs56084453	A	G	0.014935	0.002412	5.90E-10	TRUE	38.35178
rs6793835	G	A	-0.01299	0.002238	6.60E-09	TRUE	33.66108
rs13084230	C	T	-0.01356	0.00246	3.50E-08	TRUE	30.39524
rs4833095	T	C	0.025111	0.002406	1.70E-25	TRUE	108.9646
rs13122455	C	T	-0.014	0.002464	1.30E-08	TRUE	32.29318
rs62370429	T	C	-0.02616	0.004348	1.80E-09	TRUE	36.19898
rs142169179	G	A	0.040185	0.00734	4.40E-08	TRUE	29.97094
rs2248162	T	C	0.011819	0.002047	7.80E-09	TRUE	33.33078
rs9640029	C	T	-0.0138	0.001974	2.70E-12	TRUE	48.89785
rs140295641	T	A	-0.03514	0.006172	1.30E-08	TRUE	32.40604
rs2004679	T	C	0.011915	0.002138	2.50E-08	TRUE	31.04924
rs61620752	T	G	0.016069	0.002767	6.40E-09	TRUE	33.71969
rs61875074	A	C	0.022266	0.003841	6.80E-09	TRUE	33.6006
rs147895162	T	C	-0.04476	0.008107	3.40E-08	TRUE	30.48071
rs10498638	T	C	0.014014	0.002537	3.30E-08	TRUE	30.51287
rs74247887	C	T	0.037132	0.005881	2.70E-10	TRUE	39.86984
rs74805019	G	C	-0.03071	0.005482	2.10E-08	TRUE	31.38607
rs4788565	G	A	-0.02192	0.003994	4.10E-08	TRUE	30.11083
rs7200852	C	A	-0.02443	0.00445	4.00E-08	TRUE	30.12939
rs60304336	G	T	0.027932	0.00503	2.80E-08	TRUE	30.84191

Supplementary Table 2: The instrumental variables of PM10.

SNP	other_allele	effect_allele	beta.expos	se.exposure	pval.expos	mr_keep.e	f
rs76170056	C	A	0.016508	0.003462	1.90E-06	TRUE	22.73626
rs71323440	C	T	0.016293	0.003408	1.70E-06	TRUE	22.85868
rs9997134	T	C	-0.01047	0.002255	3.40E-06	TRUE	21.56995
rs138141967	G	T	-0.04294	0.009155	2.70E-06	TRUE	21.99721
rs116816317	G	A	0.037649	0.007965	2.30E-06	TRUE	22.34258
rs13125748	C	A	0.011453	0.002502	4.70E-06	TRUE	20.95909
rs116259145	C	A	0.030164	0.006185	1.10E-06	TRUE	23.78444
rs78060907	C	A	-0.03805	0.007698	7.70E-07	TRUE	24.43949
rs9497937	C	A	-0.01277	0.002577	7.20E-07	TRUE	24.55559
rs111308789	T	A	-0.02194	0.004729	3.50E-06	TRUE	21.52391
rs17675316	A	G	0.036905	0.00788	2.80E-06	TRUE	21.93191
rs1706918	G	A	0.018349	0.003677	6.00E-07	TRUE	24.90639
rs118101191	G	T	0.059791	0.011063	6.50E-08	TRUE	29.20813
rs57048268	A	C	-0.01102	0.002365	3.20E-06	TRUE	21.70542
rs1157546	T	C	-0.0246	0.004901	5.20E-07	TRUE	25.20478
rs117389221	T	C	-0.04022	0.008501	2.20E-06	TRUE	22.38495
rs605027	C	T	-0.01139	0.002454	3.40E-06	TRUE	21.56452
rs8006373	T	A	-0.01758	0.003778	3.30E-06	TRUE	21.65915
rs11621531	G	A	-0.02313	0.004487	2.50E-07	TRUE	26.56476
rs10152521	T	C	0.114394	0.023431	1.00E-06	TRUE	23.83512
rs8051340	C	G	0.016073	0.003326	1.30E-06	TRUE	23.35649
rs62079137	T	C	-0.01735	0.003743	3.60E-06	TRUE	21.49039
rs12462492	G	T	-0.01239	0.002496	6.90E-07	TRUE	24.64175
rs117125329	C	G	0.055167	0.011992	4.20E-06	TRUE	21.16166

Supplementary Table 3: The instrumental variables of PM2.5-10.

SNP	other_allele	effect_allele	beta.expos	se.exposure	pval.expos	mr_keep.e	f
rs62180536	C	T	-0.0321	0.006078	1.30E-07	TRUE	27.88863
rs73072692	C	T	0.012482	0.002413	2.30E-07	TRUE	26.7514
rs974801	A	G	0.010699	0.002102	3.60E-07	TRUE	25.90722
rs76682037	A	G	-0.04941	0.009828	5.00E-07	TRUE	25.27641
rs12203592	C	T	0.015941	0.002408	3.60E-11	TRUE	43.82627
rs79475047	T	C	0.032731	0.006197	1.30E-07	TRUE	27.89292
rs2517897	C	A	0.014476	0.002814	2.70E-07	TRUE	26.45799
rs62458757	C	G	-0.02912	0.005628	2.30E-07	TRUE	26.7657
rs6954825	T	C	0.014131	0.002765	3.20E-07	TRUE	26.12122
rs77205736	C	T	0.015406	0.002269	1.10E-11	TRUE	46.11078
rs2926098	C	T	-0.01907	0.003709	2.70E-07	TRUE	26.45243
rs10983735	G	A	0.016311	0.002803	5.90E-09	TRUE	33.86681
rs11191447	C	T	0.020186	0.003733	6.40E-08	TRUE	29.2323
rs34623735	C	T	0.01272	0.002157	3.70E-09	TRUE	34.7748
rs149827261	G	T	-0.01501	0.00292	2.70E-07	TRUE	26.4304
rs4105558	C	G	-0.01144	0.002181	1.60E-07	TRUE	27.50886
rs9541967	T	C	-0.01611	0.003077	1.60E-07	TRUE	27.41349
rs35731545	C	T	-0.03921	0.007774	4.60E-07	TRUE	25.43817
rs62062033	C	T	0.014352	0.002719	1.30E-07	TRUE	27.85642
rs7225402	T	C	-0.02489	0.004315	8.00E-09	TRUE	33.27316
rs2290154	T	C	0.011101	0.002116	1.50E-07	TRUE	27.53606
rs1978728	C	T	-0.01102	0.002108	1.70E-07	TRUE	27.32759

Supplementary Table 4: The instrumental variables of NO_2.

SNP	other_allele	effect_allele	beta.expos	se.exposure	pval.expos	mr_keep.e	f
rs6667345	C	T	0.012721	0.002456	2.20E-07	TRUE	26.82438
rs7514956	A	C	-0.01461	0.002652	3.60E-08	TRUE	30.34155
rs12089815	G	A	-0.01062	0.002079	3.30E-07	TRUE	26.0759
rs6749467	G	A	-0.01166	0.002096	2.60E-08	TRUE	30.95781
rs10172295	G	A	-0.01133	0.002099	6.80E-08	TRUE	29.10981
rs13429081	A	T	0.013454	0.002661	4.30E-07	TRUE	25.55829
rs144720952	A	G	0.04988	0.009756	3.20E-07	TRUE	26.1392
rs1318845	T	C	-0.01417	0.002596	4.80E-08	TRUE	29.80228
rs17513184	C	T	-0.01994	0.003855	2.30E-07	TRUE	26.7465
rs27152	C	T	-0.01187	0.002211	8.00E-08	TRUE	28.81844
rs72808024	A	C	-0.01703	0.002906	4.60E-09	TRUE	34.33572
rs12203592	C	T	0.01937	0.002457	3.20E-15	TRUE	62.16427
rs77255816	C	T	0.029905	0.005471	4.60E-08	TRUE	29.87975
rs115860766	C	T	0.02001	0.003837	1.80E-07	TRUE	27.19761
rs7776279	G	A	0.01165	0.002292	3.70E-07	TRUE	25.83019
rs34969378	G	A	-0.01132	0.002225	3.60E-07	TRUE	25.88912
rs73085844	T	G	-0.03311	0.006535	4.00E-07	TRUE	25.67701
rs77205736	C	T	0.013265	0.002315	1.00E-08	TRUE	32.84429
rs1217106	A	G	0.014557	0.002519	7.50E-09	TRUE	33.40472
rs118091313	G	A	0.09028	0.017779	3.80E-07	TRUE	25.78369
rs3740390	C	T	0.020166	0.003811	1.20E-07	TRUE	28.0016
rs11049241	C	A	0.013702	0.002654	2.40E-07	TRUE	26.64927
rs11855821	G	A	-0.01184	0.002336	4.00E-07	TRUE	25.68451
rs8614	C	A	0.013909	0.002677	2.00E-07	TRUE	26.99609
rs17183854	T	G	-0.04794	0.009091	1.30E-07	TRUE	27.81482

Supplementary Table 5: The instrumental variables of Nox.

Exposure	No. SNPs	F(min)	P (Q test)	P(MR-Egger intercept test)	P(MR-PRESSO global test)
PM2.5	10	25.869	0.394	0.975	0.585
PM10	18	29.971	0.179	0.009	0.249
PM2.5-10	19	20.959	0.035	0.77	0.141
Nitrogen dioxide	17	25.276	0.395	0.061	0.187
Nitrogen oxides	17	25.558	0.144	0.749	0.183

Supplementary Table 6: Sensitivity test of MR analysis results.

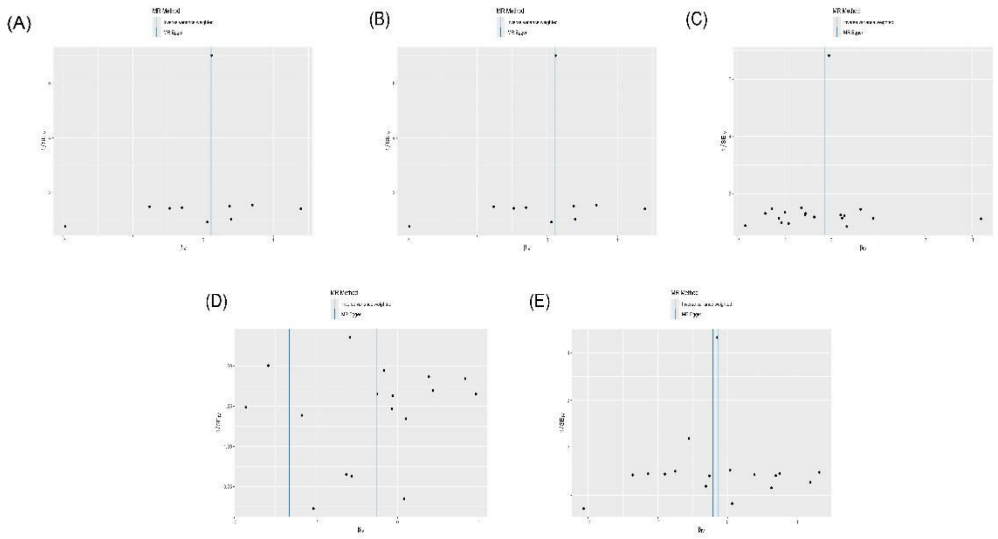

Supplementary Figure 1: Funnel plot showing the relationship between the overall effect estimate of air pollution on migraine and the inverse standard error of individual SNP effect estimates. Points represent SNPs, and the two vertical lines indicate the overall effect values calculated by two different methods. The distribution of points reflects the heterogeneity of the analysis. (A) PM2.5; (B) PM10; (C) PM2.5-10; (D)Nitrogen dioxide; (E)Nitrogen oxides
